# Controlling Pickering Emulsion Destabilisation: A Route to Fabricating New Materials by Phase Inversion

**DOI:** 10.3390/ma9080626

**Published:** 2016-07-27

**Authors:** Catherine P. Whitby, Erica J. Wanless

**Affiliations:** 1 Institute of Fundamental Sciences, University of Massey, Palmerston North 4410, New Zealand; 2 Priority Research Centre for Advanced Particle Processing and Transport, University of Newcastle, Callaghan, NSW 2308, Australia; Erica.Wanless@newcastle.edu.au

**Keywords:** Pickering emulsion, particle-stabilised emulsion, destabilisation

## Abstract

The aim of this paper is to review the key findings about how particle-stabilised (or Pickering) emulsions respond to stress and break down. Over the last ten years, new insights have been gained into how particles attached to droplet (and bubble) surfaces alter the destabilisation mechanisms in emulsions. The conditions under which chemical demulsifiers displace, or detach, particles from the interface were established. Mass transfer between drops and the continuous phase was shown to disrupt the layers of particles attached to drop surfaces. The criteria for causing coalescence by applying physical stress (shear or compression) to Pickering emulsions were characterised. These findings are being used to design the structures of materials formed by breaking Pickering emulsions.

## 1. Introduction

Controlling emulsion stability during their storage and use is a major challenge [1,2,3,4]. Emulsions are used in cosmetic products, detergents and foods, as well as for liquid extractions and oil recovery [1,2,3]. Products like moisturizer creams take advantage of how emulsions yield and flow, their texture and visual appearance. These properties depend on the volume fraction and size of the droplets in the emulsion. They change over time due to Ostwald ripening, flocculation and coalescence of the drops.

Making an emulsion that will not age irreversibly while it is being handled requires addition of stabilising components. They can be surfactant molecules, polymers, proteins, or particles. The topic of this review is emulsion stability in the presence of particles. Ramsden [5] (and later Pickering [6]) first described the presence of a membrane of solid particles (proteins or other precipitated colloids) enhancing the lifetime of oil droplets and air bubbles in water. Recent progress has improved our understanding of the mechanisms by which solid particles slow Ostwald ripening and coalescence [4,7,8].

The remarkable stability of Pickering emulsions is a problem for applications that require controlled destabilisation of emulsions. Particle-stabilised emulsions that form during the extraction of bitumen from oil sands, for example, are difficult to break. They reduce the volume of oil recovered and generate waste (the unwanted emulsion) [9,10,11]. Pickering emulsion formation during biphasic reactions catalysed by nanoparticles increases the reaction yield, but reduces its efficiency [12,13]. They hinder separation of the products from the reaction mixture and recycling of the catalyst [14,15]. Particle separations using biphasic extractions are also hindered by particles becoming trapped at the liquid interface [16,17]. 

Emulsions and foams are destabilised to make coatings and adhesives by evaporating the volatile components to leave a film of active ingredients on a solid surface. Using Pickering emulsions as precursors for assembling films of particles on surfaces, for example, relies on the particle-coated drops or bubbles coalescing with a flat oil-water or air-water interface [18,19]. Although Pickering emulsions are templates for assembling particles into porous solids [20,21,22], the particle networks tend to collapse during drying [23,24]. The voids in the solids formed typically lack the desired polyhedral geometry. 

The focus of this review is on developments in our understanding of how particle-stabilised emulsions break down. Denkov et al. [25] first proposed that Pickering emulsions destabilise if there are defects, like fractures or vacancies, in the particle layer coating the drops. The defects cause the thin films separating drops to rupture and the drops coalesce. One approach to controlling Pickering emulsion stability is to synthesise particles that respond to an external stimulus by detaching from the drop surface. The particles are used to form emulsions that can be destabilised on demand. This approach was recently reviewed comprehensively [26]. Here we focus on the structural changes that occur in Pickering emulsions as they age. We describe how emulsions are broken by particle detachment, mass transfer and drop coalescence. Then we discuss the materials being fabricated by harnessing destabilisation processes in Pickering emulsions. 

## 2. Detaching Particles from Fluid Interfaces

Particles (of radius *r_p_*) assemble at oil-water interfaces (of interfacial tension, γ*_ow_*) by becoming partially immersed in both liquids and forming a three phase oil-water-particle contact angle, θ*_ow_*. Attached particles reduce the total interfacial area between the oil and water. This alters the free energy of the system by changing the balance of surface energies. For spherical particles with θ*_ow_* < 90°, the free energy of attaching a particle to a drop is denoted Δ*_a_G*, and is given by [27,28]:

(1) $$\Delta_{a}G=-{\pi \gamma }_{ow}r_{p}^{2}{\left(1-cos\theta_{ow}\right)}^{2}$$

**Figure 1:**
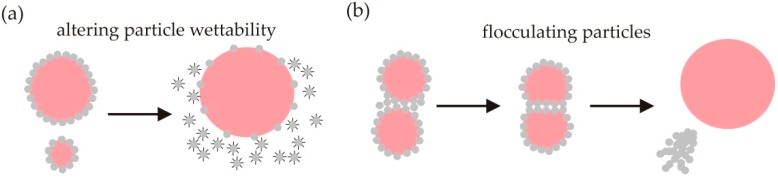
Pickering emulsions are destabilised by detaching particles from the emulsion drops by (**a**) using surfactants that adsorb to the particle surfaces and modify their wettability so that they favour being fully wetted by the continuous phase; or (**b**) enhancing particle flocculation sufficiently to favour adhesion between drops and rupture of the interfacial particle layers which produces flocs of particles and uncoated drops of the dispersed phase.

The interfacial energy trapping a particle at an oil-water interface calculated using Eqn 1 is significant for particles on the colloidal length scale (up to 10^4^ *kT* for *r_p_* ~ 10 nm). The lack of thermodynamic stability in Pickering emulsions arises because bare oil-water interface remains between the particles attached to the drop surfaces. The positive free energy of forming this interface always outweighs the negative contribution from particle attachment. Although attached particles are not at equilibrium, particles with radii larger than several nanometres attach to oil-water interfaces effectively irreversibly, giving emulsions kinetic stability. In this section we discuss destabilising emulsions by altering the particle wettability or by competitive displacement with surfactant molecules and by modifying the particle flocculation (Figure 1).

### 2.1. Altering Particle Wettability

Attached particles can be displaced by using surfactants that adsorb to the particle surfaces and modify their wettability in situ (Figure 1a) [29,30,31]. Alargova et al. [29] caused particles to detach from foams stabilised by hydrophobic polymer microrods by gently adding a few drops of a concentrated anionic surfactant (sodium dodecyl sulfate, SDS) solution. They argued that adsorption of SDS onto the particle surfaces made them hydrophilic and caused them to detach from the air–water interface. As the foam collapsed, the particles drained from the foam into the lower aqueous phase. Subramanian et al. [31] observed that isolated bubbles (∼100 μm in diameter) stabilised by micrometre-sized latex particles became unstable to disproportionation and particle detachment after exposure to SDS or Triton X-100 (non-ionic surfactant). They also argued that at high surfactant concentrations, the surfactant adsorbed onto the particle surfaces and made them hydrophilic. 

How the wettability of micrometre-sized polystyrene latex particles at a planar decane-water interface is altered by adding SDS to the water was investigated by Reynaert et al. [30]. They suggested that altering θ*_ow_*, and hence the extent to which the particles are immersed in each liquid, will affect the interaction forces between particles at the interface. Their examination of the particle arrangement in the interfacial layer revealed that the latex particles assembled into network structures that were looser (more open) in the presence of SDS [30]. They showed that the contact angle of a drop of water on a polystyrene film immersed in decane increased as the concentration of SDS increased in the water and argued that this was due to adsorption of SDS [30]. Moreover they argued that adsorption of SDS onto latex particles attached to a decane-water interface must increase the oil wettability of the particles and hence weaken the lateral capillary interaction forces between the particles.

### 2.2. Competitive Displacement of Particles

Another strategy for displacing particles from drop surfaces is to add surfactant which competes for the oil-water interface [32,33,34]. Vella et al. [35] observed that adding surfactant could disrupt particle layers attached to planar water surfaces. They examined densely packed monolayers of polymeric particles (*r_p_* = 50 μm) on the surfaces of water-glycerol solutions. They used a needle to inject a drop of non-ionic surfactant (polyoxyethylene sorbitan monoleate) into the layer. Providing the particles were not jammed together, Vella et al. [35] observed a crack form, where the needle touched the particles, and propagate through the monolayer. They argued that localised reduction of the surface tension caused tensile stress in the particle layer, forcing the particles to rearrange [35]. As the crack propagated, the particles consolidated and exposed the liquid surface. Once the particles jammed, they trapped the crack in its final shape for several hours.

**Figure 2:**
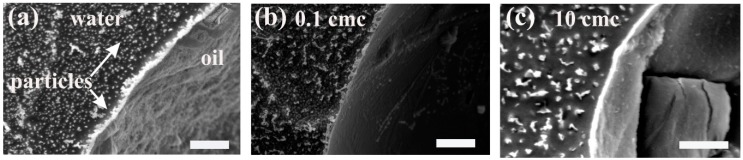
Electron microscopy images of the oil-water interface in a Pickering emulsion mixed with surfactant at concentrations of (**a**) 0 M; (**b**) 10% of the critical micelle concentration; and (**c**) ten times the critical micelle concentration [34]. The layer of densely packed particles at the drop surfaces is disrupted by surfactant adsorption at concentrations below the critical micelle concentration. The particles are completely displaced from the interface at surfactant concentrations above the critical micelle concentration. The scale bars correspond to 2 μm in the left and middle images and 1 μm in image on the right. Adapted from [34] with permission from Elsevier.

Vashisth et al. [34] showed that mixing dodecane-in-water emulsions stabilised by silanised fumed silica nanoparticles (*r_p_* ~ 10 nm) with solutions of anionic surfactant (SDS) causes the nanoparticles to be displaced from the drop surfaces. Rather than adsorbing onto the particle surfaces (which are already coated with hydrocarbons), the surfactant adsorbs competitively at the oil-water interface. Two minutes of mechanical mixing was required to completely displace the particles from the drop surfaces after adding surfactant at concentrations above the critical micelle concentration [34]. Examination of drop surfaces in emulsions mixed with lower surfactant concentrations revealed that the drops were coated with a mixed layer of nanoparticles and surfactant (Figure 2) [34]. They speculated that particle displacement occurs by a mechanism similar to the displacement of proteins [36] by surfactants. Like particles, proteins stabilise interfaces by forming an immobile, viscoelastic film. Adding surfactant to protein-stabilised emulsions and stirring can induce displacement of proteins from the drop surfaces. This was linked to the reduction of the interfacial tension caused by surfactant adsorption [37]. 

Katepalli et al. [33] argued that surfactant addition will cause particle displacement from a drop (of radius, *r_d_*) if a surfactant-stabilised drop (of the same radius) has a lower free energy than the particle-stabilised drop. Thus adding surfactant causes particle displacement due to the emulsion system seeking a lower energy state. Katepalli et al. [33] found that for the surfactant-stabilised emulsion to be more stable, the following inequality must be satisfied:

(2) $$\frac{\gamma_{ow}-\gamma_{os}}{\gamma_{ow}}>f{\left[\frac{1-cos\theta_{ow}}{\sin\theta_{ow}}\right]}^{2}$$

where γ*_os_* is the interfacial tension of a surfactant-stabilised drop and *f* is the fraction of the interfacial area occupied by particles. For particles of intermediate (or neutral wettability (θ*_ow_* = 90°), surfactant addition can cause particle displacement if the fractional change in the oil-water interfacial tension with surfactant adsorption is greater than the fraction of the drop surfaces coated with particles. Katepalli et al. [33] examined the response of octane-in-water emulsions stabilised by carbon black nanoparticles to exposure to solutions of surfactant at their critical micelle concentrations. They found that adding Triton X-100, which reduces the interfacial tension by 94% from 51 to 3 mN·m^−1^, was sufficient to displace carbon black particles from the octane drops [33]. In contrast, adding sodium octyl sulfate only reduced the interfacial tension by 67%, which was not sufficient to cause particle displacement. 

### 2.3. Flocculating Drops and Particles

Particle-stabilised emulsions are also sensitive to flocculation of the particles. Briggs [38] and Lucassen Reynders and van den Tempel [39] first reported that weakly flocculated particles are most efficient at (kinetically) stabilising emulsions. Briggs [38] argued that this was due to strong flocculation producing particle aggregates which are too large to assemble into layers at the surfaces of drops. 

Horozov and Binks [40] showed that there is the potential for particle-coated drops to flocculate by particle bridging, where particles attach simultaneously to two drop surfaces. They proposed that bridging occurs where there are strong repulsive interactions between the particles and they form dilute monolayers at the oil-water interface [40]. Bridging occurs when particles on opposing interfaces interlock as the interfaces come together [41,42]. French et al. [43] demonstrated that for particle bridging to occur, there must be insufficient particles present to stabilise the interfacial area in the emulsion and the particles must be preferentially wet by the continuous phase.

Binks and Lumsdon [44] found that drops flocculate in emulsions formed under conditions corresponding to the onset of particle flocculation. Horozov et al. [45] proposed that the drops form three dimensional networks with the particles at the onset of particle flocculation. Evidence of network formation in Pickering foams was found by Chuanuwatanakul et al. [46]. They observed that foams stabilised by mixtures of nanoparticles and surfactants had a granular morphology at surfactant concentrations sufficient to cause strong flocculation of the particles [46]. Subsequently Whitby et al. [47] showed that the energy of adhesion between the particle layers coating the drops increases as the extent of particle flocculation increases. Coalescence is favoured under conditions of strong particle flocculation, where the adhesive energy between the particles is comparable to the energy required to detach the particles from the drops (Figure 1b) [47].

## 3. Transferring Mass between the Liquid Phases

**Figure 3:**
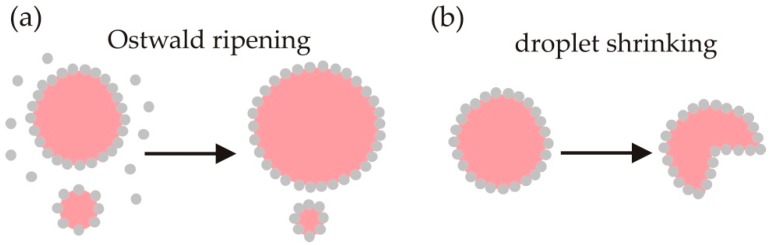
Pickering emulsions destabilise by mass transport due to (**a**) Ostwald ripening with small drops shrinking and larger drops swelling as molecules transfer from the small to the larger drops; and (**b**) all drops shrinking due to the liquid phases in the emulsion becoming partially miscible. The layer of particles coating shrinking drops buckle when the particles become jammed.

Pickering emulsions can destabilise by the transfer of mass between drops of different sizes, or between drops and the continuous phase (Figure 3). The former process is known as Ostwald ripening and causes a fraction of the drops to increase in size. In the latter process the average drop volume is reduced (or shrunk) by causing the liquid in the drops to dissolve or evaporate.

### 3.1. Ostwald Ripening

One of the mechanisms by which drops coarsen in oil-in-water Pickering emulsions (and air-in-water foams) is Ostwald ripening. It is controlled by the molecular solubility of the oil (or air) in the aqueous phase. Molecules in small drops transfer to larger drops due to the difference in Laplace pressure across the fluid interfaces of differently sized drops (Figure 3a). Particles attached to the interfaces in emulsions and foams can arrest Ostwald ripening. Ashby and Binks [48] found that Ostwald ripening in toluene-in-water emulsions formed in the presence of laponite nanoparticles is initially rapid and then ceases at long times. They proposed that particles in the continuous phase may attach to the freshly created surfaces of the growing drops, as illustrated in Figure 3a. Since the clay particles attached to the drops are not easily displaced, they compress into an insoluble barrier around the shrinking drops and eventually halt ripening [48]. Cates [49] argued that Pickering emulsions resist Ostwald ripening if the particles are jammed around the surfaces of shrinking drops, because they cannot follow the drop surface inwards. Instead the interface develops in such a way as to have zero mean curvature. The Laplace pressure thus drops to zero and Ostwald ripening is halted.

That bubbles coated with micrometre-sized latex particles deform over time into polyhedral shapes, with flattened faces and rounded edges and corners was shown by Abkarian et al. [50]. They used simulations to show that these bubble shapes are stable to disproportionation, since this is a minimum energy configuration and the Laplace pressure across the flattened fluid interface is negligible [50]. Meinders and van Vliet [51] used numerical simulations to show that Ostwald ripening in a Pickering emulsion is arrested if particles attached to the drops cause their surfaces to resist compression, and the surface elastic compression modulus (*E*) is at least twice the surface tension. Later Cervantes Martinez et al. [52] showed experimentally that the condition for stability to Ostwald ripening in a particle-stabilised foam is that *E* > γ*_aw_*/2.

Attached particles can fail to arrest Ostwald ripening. Ettelaie and Murray [53,54] argued that the rate of bubble dissolution in foams can be faster than the rate at which particles are transported to bubbles and attach to their surfaces. They calculated that the bubble size distribution broadens with time in the case where the particle concentration is higher than that required to stabilise the total air-water interface, since it is governed by the time taken for particle attachment [53,54]. For cases where the particle concentration is not sufficient to stabilise the air-water interface, the bubble size distribution narrows, as the final interfacial area is determined by the number of particles available [53]. 

The rate of Ostwald ripening in emulsions is slower than the rate of particle attachment to the drop surfaces. In the case where there are insufficient particles to stabilise the total oil-water interface in emulsions, Avendano Juarez and Whitby [55] showed experimentally that destabilisation initially occurs by a combination of droplet flocculation and ripening. Close contact between the flocculated drops enhances oil transfer from smaller drops to larger ones [55]. Large drops swell over time until the density of attached particles is insufficient to protect the drops against coalescence [55].

When two different o/w emulsions containing mutually miscible oils are mixed, mass transfer between the droplets can produce a single population of drops containing a mixture of the oils. This process is called compositional ripening. It is related to Ostwald ripening, however the chemical potential difference due to the concentration differences normally outweighs that due to Laplace pressure differences, and mass transfer is dominated by compositional ripening. Binks et al. [56] showed that compositional ripening in mixtures of Pickering emulsions triggers droplet coalescence, unlike in surfactant-stabilised emulsions where the drops swell, but do not coalesce. They found that adding excess particles suppressed the swelling-triggered coalescence as the particles attach to and stabilise the fresh oil-water interface being created [56]. If coalescence was not suppressed, the merging drops tended to become trapped in non-spherical shapes (this is known as arrested coalescence behavior and is discussed later).

### 3.2. Shrinking Drops

Where the liquid phases used to form an emulsion become partially miscible, the emulsion can destabilise by droplet shrinking (Figure 3b). Many pairs of immiscible liquids, for example, begin to mix when heated or cooled. Clegg et al. [57] used confocal fluorescence microscopy to visualise drop shrinking in particle-stabilised oil-in-alcohol emulsions as they were slowly warmed up to the temperature where the alcohol and oil formed a single liquid phase (the upper critical solution temperature). They observed that the particle-laden drop surfaces buckled and cracked, and argued that the cracks allow the liquid inside the drops to leave and mix with the external liquid [57].

**Figure 4:**
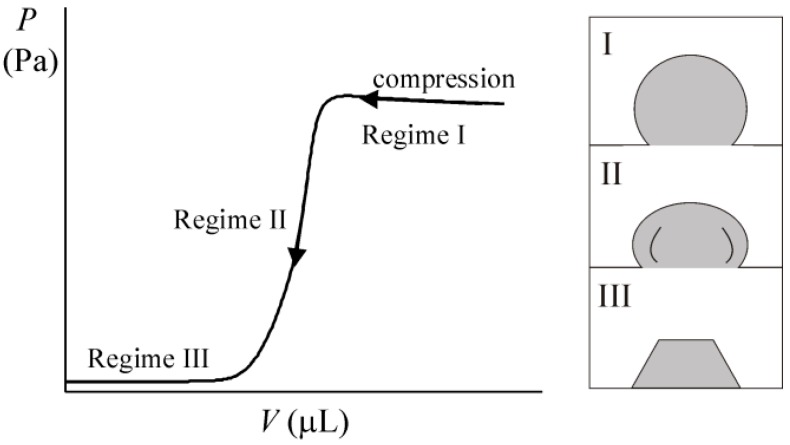
Behaviour of a drop coated with particles as the drop volume is reduced [60]. The pressure inside the drop increases slightly for only small reductions in the drop volume (Regime I). The interface remains fluid-like and the drop profile shrinks isotropically. In Regime II, the particles pack closely together and jam. The pressure inside the drop falls to zero and the drop takes on the shape of a wrinkled hemisphere. At even larger reductions in drop volume (Regime III), the pressure is insensitive to the compression. The particle layer coating the drop buckles and flattens at the top of the drop.

The shrinking of macroscopic, pendant drops of water in silicone oil that were coated with hydrophobic silica crytals (*r_p_* ~ 6.5 μm, θ*_ow_* = 125^o^) was visualised by Asekomhe et al. [58]. As water was sucked out of the drops, they lost their spherical shape and buckled [58]. Datta et al. [59] visualised the changes in shape of particle-coated drops in emulsions where the internal phase was slightly soluble in the external phase. They found that an increasing proportion of the drops buckle as the drop volume was systematically reduced. Larger drops buckled more easily than smaller drops [59]. The shrunken drops resembled buckled structures formed by solid shells under compressive stress. These observations supported their hypothesis that densely-packed layers of colloidal particles at fluid interfaces act collectively like solid layers [59]. By measuring the pressure in particle-coated droplets as they were deflated, Xu et al. [60] demonstrated that there is a transition from fluid-like to solid-like behaviour in the particle shell as it is compressed, as shown in Figure 4.

Aveyard and co-workers [61,62] examined the compression of layers of particles at the planar air-water surface of a Langmuir trough. They demonstrated that when a layer of particles at a planar fluid interface is compressed, it bends and forms an undulating surface with a characteristic wavelength. Wrinkling occurs because the area occupied by the attached particles remains constant although the area of the trough has decreased. Lateral compression causes the coating to expand (wrinkle) in the perpendicular directions. Following the general theory for wrinkling of elastic sheets, Vella et al. [63] showed that the periodicity (λ) of the wrinkles in particle coatings can be estimated [63] by:

(3) $$\lambda =\pi {\left[\frac{4}{3\left(1-\varphi \right)\left(1+\nu \right)}\right]}^{1/4}\sqrt{L_{c}r_{p}}$$

where ϕ is the area fraction of particles at the liquid surface, ν is the Poisson ratio of the particle shell and *L_c_* is the capillary ratio of the drop. Whitby et al. [64] found that the wavelength in crumpled particle layers on coalesced drops is consistent with that predicted by Equation (3).

Razavi et al. [65] investigated the mechanisms by which planar liquid surfaces laden with particles collapse as they are compressed. They used a Langmuir trough to study the surface pressure of air-water surfaces coated with close-packed monolayers of silica spheres (*r_p_* = 500 nm) modified to different extents by reaction with dichlorodimethylsilane. Relatively hydrophilic particles formed a fluid-like monolayer that experienced an irreversible collapse. Microscopy images of the surface revealed that this was likely due to expulsion of the particles into the water [65]. In contrast, more hydrophobic particles formed a solid-like, cohesive monolayer that exhibited a prominent compressional elasticity through reversible wrinkling and folding [65]. Stress relaxation was arrested by some of the hydrophobic particles ejecting into the aqueous subphase. Razavi et al. [65] argued that particle-laden oil-water surfaces might show different collapse behavior, due to the long-range forces [66] between the remaining particles that are mediated by the oil phase. Garbin et al. [67] visualised the contraction of pendant oil drops coated with gold nanoparticles in water. The nanoparticles detached from the surface as the drop volume decreased and the particles became close-packed. Furthermore, these workers suggested that short-range steric repulsions between the ligand-capped particles played a crucial role in detachment.

## 4. Coalescing Drops

**Figure 5:**
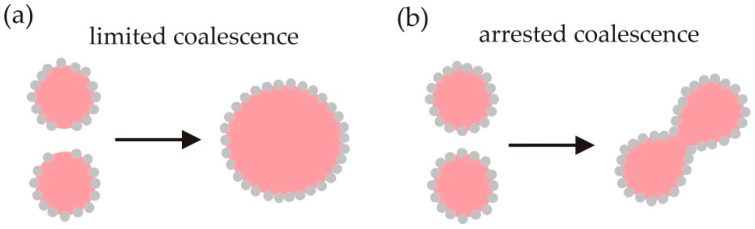
Pickering emulsions destabilise by (**a**) limited coalescence until a critical surface coverage of particles is reached if there are not sufficient particles present to fully coat all the drop surfaces that form initially; and by (**b**) arrested coalescence where the combined surface area occupied by particles is higher than the interfacial area that would form by complete coalescence of the drops, so the coalescing drops remain trapped in an intermediate stage of coalescence, unable to relax into a spherical shape.

Coalescence is a process in which two drops merge to form a larger drop. It reduces the total interfacial area in the emulsion. Coalescence occurs even during emulsion formation and must be (temporarily) halted to impart kinetic stability to an emulsion. Figure 5 shows that the strong (irreversible) attachment of particles to interfaces means that coalescence can be limited or arrested in Pickering emulsions, unlike in surfactant-stabilised foams or emulsions.

### 4.1. Limited Coalescence During Emulsion Formation

Arditty et al. [68] found that if, during emulsion formation, the total number of particles present is not sufficient to fully coat the oil-water interface, the drops will coalesce together until a critical degree of surface coverage by the particles is reached (Figure 5a). By following the evolution of the drop size distribution in emulsions of millimetre-sized drops with a digital camera, they showed that the transient and final drop size distributions are relatively narrow. For a given degree of coverage of the drop surfaces by particles, τ, there is a linear relationship [68] between the average drop radius at any time (*r_d_*(*t*)) and the mass of particles (*m_p_*) which is given by:

(4) $$\frac{1}{r_{d}\left(t\right)}=\frac{s_{f}m_{p}}{\tau 3V_{d}}$$

where *s_f_* is the droplet surface area covered per unit mass of particles and *V_d_* is the volume of the dispersed phase. This is a generalisation of the relation originally proposed by Wiley [69] to account for observations that the coalescence rate in emulsions containing finely divided solids decreases to zero as the drops approach a limiting size and a relatively uniform size distribution. Fritgers et al. [70] used numerical simulations of the final states formed by a mixture of immiscible fluids and particles to confirm the dependence between the particle concentration and the average drop radius in Pickering emulsions. 

Daware and Basavaraj [71] showed that the limited coalescence model can be used to predict the drop size in emulsions formed by using micrometre-sized silica rods to arrest the temperature-induced phase separation of critical mixtures of 2,6 lutidine and water. The model has also been applied to w/o emulsions stabilised by asphaltenes. Pauchard et al. [72] observed that water drops stabilised by asphaltenes will grow until a critical mass coverage of the drop surfaces is reached and argued that this is reminiscent of limited coalescence in Pickering emulsions. They proposed that asphaltenes behave like nanoparticles and jam together into a dense, glassy monolayer at the drop surfaces [72,73].

The limited coalescence model assumes that all the particles in an emulsion are equally effective at stabilising drops. This may not be the case, however, for mixtures of different particles. In the case of oppositely charged particles, it is necessary for the particles to heteroaggregate into networks or clusters to stabilise emulsions. Whitby et al. [74] studied emulsions formed in the presence of mixtures of oppositely charged titania and silica nanoparticles. The titania particles were partially hydrophobic and, on their own, attached strongly to the oil–water interface and stabilised emulsions. The silica particles had hydrophilic surfaces and were poor emulsifiers. Adding silica particles to the titania dispersions enhanced coalescence processes during emulsion formation. This was demonstrated by Whitby et al. [74] using cryogenic scanning electron microscopy to visualise the drop surfaces. They linked the destabilisation to the presence of silica particles in the particle layers at the drop surfaces. Nallamilli et al. [75] modified the limited coalescence model to describe emulsions containing a binary mixture of oppositely charged particles. They successfully predicted the drop size dependence on the number ratio of particles in the mixed system [75].

### 4.2. Coalescence of Partially-Coated Drops after Emulsion Formation

Emulsions can form with droplets that are only partially covered by particles. Strongly repulsive colloidal particles, which form ordered, dilute planar monolayers at liquid interfaces, can act as effective emulsion stabilisers. The particles bridge the thin films between the drops in close contact. French et al. [43] showed that shearing dilute emulsions of fully-coated oil drops in water could cause particle bridging, by creating more oil-water interfacial area than could be stabilised by the available particles. Similarly, Zhang et al. [76] observed that bridging occurred in emulsions formed by ultrasonication, rather than vortex mixing, due to the larger amount of agitation creating a larger oil-water interfacial area. These emulsions become sensitive to coalescence when the repulsive interactions between the particles are enhanced, or the drops are concentrated together.

Xu et al. [77] found that hexadecane drops formed in aqueous dispersions of polydopamine particles (*r_p_* = 192 nm) were only partially coated by particles at acidic pH. Rather than forming a continuous layer, the polydopamine particles occupied segregated regions on the drop surfaces. They showed that lowering the pH in the emulsions caused the drops to coalesce together and form densely coated drops [77]. 

Traditional methods of emulsification make it difficult to systematically vary the density of nanoparticles at drop surfaces, as they involve simultaneously forming and fragmenting drops in the presence of particles. For a fixed energy input, the final drop size will vary with the particle concentration, while the particle density at the interface remains constant [68]. Microchannel emulsification offers the advantage of forming monodisperse drops where the size and particle loading of the drops are independently controlled [78]. Drop formation at a flow focusing nozzle occurs so quickly, that the drops must be equilibrated with particles to allow time for attachment to occur. Priest et al. [78] found that dodecane drops formed in microchannels and incompletely covered by silanised fumed silica particles coalesced when they were concentrated together. Similarly, Manga et al. [79] showed that emulsions are unstable to coalescence when drops are formed using rotational membranes without allowing sufficient time for particle attachment to occur. 

Fan and Striolo [80] used dissipative particle dynamics simulations to study coalescence of oil-in-water and water-in-oil drops as the density of nanoparticles on their surface was varied. The maximum force and the corresponding drop separation in the force-distance profiles during coalescence were taken as the threshold for coalescence. Their analysis [80] identified the conditions under which coalescence occurred between drops that were partially coated by nanoparticles. These included when the nanoparticles were poorly wetted by the continuous phase, and when the nanoparticles were strongly attracted to each other, or to the approaching drop surface. 

### 4.3. Coalescence Dynamics

**Figure 6:**
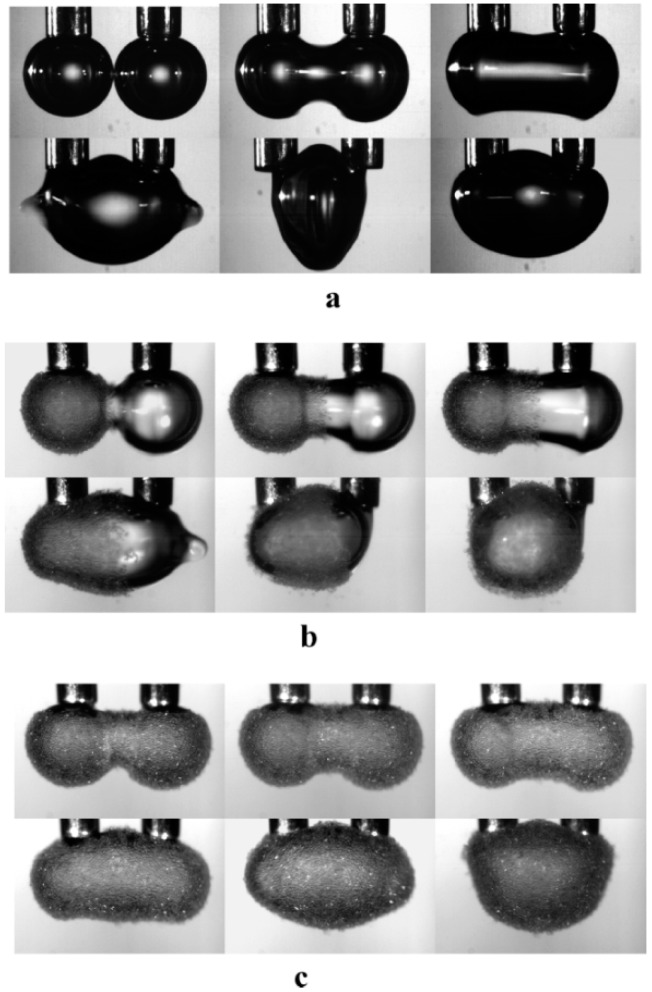
Coalescence behaviour of pairs of bubbles of equal volume generated at the tips of adjacent capillaries (with diameters of 1.07 mm). The time between the photographs is about 0.5 ms. (**a**) Bubbles with pristine surfaces initially oscillate, as the neck that forms between them extends as an expansion wave along the surface of the merging bubble. The merged bubble relaxes into a spherical shape within a few milliseconds; (**b**) In the case where one bubble is coated with silanised glass beads (with a median radius of 33 μm), the oscillations of the merging bubbles drive the beads into the centre of the bubble surface. They form a belt around the bubble surface, leaving each of the ends uncoated. The amplitude of the oscillations gradually decreases until the merged bubble reaches a stable stationary state; (**c**) Two fully coated bubbles coalesce together smoothly. The oscillations of the merging bubble are damped, presumably due to the attached particles making the bubble surface relatively rigid. Reprinted with permission from [81]. Copyright (2008) American Chemical Society.

Ata [81,82] developed a powerful method for visualising the coalescence dynamics of air bubbles coated with particles. She filmed bubbles at the tips of adjacent capillaries as they were allowed to grow until they came into contact. Pristine (uncoated) bubbles (and drops) coalescing together first oscillate, alternately expanding in the horizontal and vertical directions, until settling into a spherical shape, as shown in Figure 6a [83,84]. Ata [81] found that pristine air bubbles (*r_d_* = 1 mm) oscillated together for more than 40 ms. By analysing films of the projected area of bubbles coated with glass beads (*r_p_* ~ 33 μm) in the presence of a cationic surfactant (cetyltrimethylammonium bromide), Ata showed that the bubble oscillations were damped within ~ 30 ms (Figure 6b,c). Ata [82] proposed that the particles reduced the oscillation frequency by forming a semi-rigid shell around the bubble which increased the inertia of the bubble surface. By comparing the coalescence dynamics of bubbles coated with silanised glass beads of different hydrophobicity, Ata and co-workers [85,86] found that attaching particles with higher air-water contact angles made the bubble surfaces more rigid.

Detachment of particles from the bubble surfaces during coalescence of bubbles coated with glass beads (*r_p_* ~ 33 μm) in the presence of a cationic surfactant was also reported by Ata [82]. This detachment tended to occur at low surfactant concentrations, where the particles were presumably only weakly attached to the surface. Tan et al. [87] investigated particle detachment from bubbles with glass beads that were modified by reactions with silanes or esters to make them hydrophobic. It was predicted that increasing the particle hydrophobicity should reduce particle detachment. The fraction of particles ejected from the bubble surfaces did not vary, however, with the particle hydrophobicity [87]. Tan et al. [87] argued that this implies that particle detachment is dominated by the kinetic energy of the surface oscillations.

Ata et al. [85] observed that coating air bubbles with a close-packed monolayer of latex (*r_p_* = 190 nm) or anatase (*r_p_* = 100 nm) nanoparticles caused damping of the oscillations between coalescing bubbles within ~ 20 ms. The amplitude of the bubble oscillations was larger than those observed with bubbles coated with glass beads (*r_p_* ~ 33 μm [81]). This suggested that attached nanoparticles increase the bubble stiffness, but not to the same extent as observed for particles that are tens of micrometres in size [85]. Thompson et al. [88] found that cross-linking nanoparticles at the surfaces of oil drops caused a significant increase in drop resistance to coalescence.

### 4.4. Inducing Coalescence by Shear or Compressive Stress

Emulsion stability to coalescence is governed by the thin liquid films of continuous phase locked between touching drops. For a thin film to be in mechanical equilibrium, the repulsive surface forces in the film must balance the external forces pushing the drop surfaces against each other. Rupture occurs once liquid has drained out of the film sufficiently for the surfaces to come close enough for van der Waals attractions to dominate. In the case of Pickering emulsions, the energy required to detach the particles from the drop surfaces is an important contribution to the efficiency with which particles stabilise the drops (see Equation (1)). It will not, however, prevent liquid from draining out of the films. The capillary pressure arising from the deformation of the liquid interface around the attached particles as liquid is squeezed out causes the film to resist thinning and rupture. Thus causing coalescence in emulsions almost always requires deformation of drops by shear or compressive stresses. Deformation creates extra surface that is not occupied by particles. Rupture can then occur at these “weak spots” in the film.

The response of individual drops to shear or compressive stress reveals the dynamics of drop deformation [89]. Becu and Benyahia [90] studied the deformation and relaxation of individual particle-coated drops under jumps in strain imposed by a counter-rotating shearing device. Retraction of particle-coated drops was slower than the pristine drops, with the drops taking about 20 times longer to return to a spherical shape once the strain was removed [90]. Tan et al. [91] found that individual oil droplets coated with kaolinite particles and compressed by a colloidal particle (that was much larger than the drop) were mechanically robust and recovered their spherical shapes after large deformations. Russell and co-workers [92,93] investigated the effect of covalently cross-linking particles into membranes around drops. They found that cross-linked drops deform irreversibly. While the elasticity of unmodified capsules indicated that the interfacial tension did not change with the applied strain, the elastic response of the cross-linked capsules changed as the strain increased, suggesting the membranes had fractured [92]. Asare-Asher et al. [94] found that water marbles (water drops coated with hydrophobic particles that are only slightly immersed in the water) can withstand deformations of up to 30% and recover their spherical shapes. Higher deformations cause the particle layer to crack [94].

Dilute emulsions tend to yield and flow in response to shear stress. Whitby et al. [95] investigated coalescence in dilute oil-in-water Pickering emulsions (at a drop volume fraction, ϕ = 0.5 under shear applied by a rotational rheometer. The bromohexadecane drops (*r_d_* = 35 μm) were stabilised by silanised fumed silica particles (*r_p_* ~ 10 nm) that formed layers a few hundred nanometres thick around the drops. There were excess silica nanoparticles in the water which formed networks that entrapped the drops and enhanced emulsion stability at rest. At dilute ϕ, coalescence requires drops to collide and remain in contact long enough for the thin film formed between them to drain and rupture. They found that the susceptibility of the drops to orthokinetic coalescence depended on the extent of particle flocculation in the network of particle-coated drops and excess particles [95]. Moreover, they were able to successfully manipulate the particle flocculation and hence the extent of demulsification by varying the salt concentration in the aqueous phase.

Kruglyakov et al. [96] investigated coalescence in dilute Pickering emulsions (ϕ = 0.5 as they were compressed in a centrifugal field. The decane-in-water emulsions were stabilised by hydrophilic silica nanoparticles that had been modified by adsorbing cationic surfactant onto their surfaces. They found that the critical capillary pressure required to break the emulsions was significantly lower than the theoretically predicted maximum capillary pressure in a film stabilised by two layers of closely packed spherical particles [96]. 

Tcholakova et al. [4] compared the critical pressure leading to coalescence measured by Kruglyakov et al. [96] to the critical pressure values calculated for various types of emulsifiers from the available literature data on centrifugation studies of emulsion stability. They scaled the critical pressure by the inverse of the average drop radii in the emulsions. Tcholakova et al. [4] calculated that the scaled value of the critical pressure for surfactant and protein-stabilised emulsions ranges between 0.1 and 0.3 Pa·m. They estimated that the scaled value of the critical pressure measured by Kruglyakov et al. [96] was about an order of magnitude lower (assuming *r_d_* ~ 20 μm).

Concentrated Pickering emulsions can behave like solids, showing striking rigidity in response to small applied stresses. Arditty et al. [97] found that the elastic storage moduli of Pickering emulsions were significantly higher than those of surfactant-stabilised emulsions. They proposed that strong lateral attractions between attached particles make the drop surfaces extremely rigid. Arditty et al. [97] tested the coalescence stability of the emulsions by centrifuging the emulsions. They found that the magnitude of the compressive stress required to induce coalescence was consistent with that predicted using the elastic coefficient of the drop surfaces. The scaled value of the critical pressure measured by Arditty et al. [97] was about 0.3 Pa·m.

Hermes and Clegg [98] investigated the yielding behaviour of concentrated emulsions of oil drops (*r_d_* = 7.5 μm) in water stabilised by silica nanoparticles (*r_p_* = 330 nm). Adding high concentrations of salt (44.8 wt. %) caused the drops to flocculate and they saw evidence for this being due to aggregation between particles attached to neighbouring drops. The flocculated emulsion flowed once sufficient strain was applied to break the drop clusters apart. Compressing the emulsion to drop volume fractions, ϕ ~ 0.95 increased its elasticity by two orders of magnitude. At these *φ*, the rate-determining step for coalescence is rupture of the thin films between drops. The drops in the compressed emulsion coalesced instead of flowing when the emulsion yielded. Hermes and Clegg [98] argued that this was due to the particle stabilisation of the interface failing at high strains. The scaled value of the critical pressure was about 0.2 Pa·m.

### 4.5. Partial and Arrested Coalescence

Partial coalescence of drops can occur in o/w emulsions where the oil drops contain crystals, if a few crystals protrude out from the drops into the continuous water phase. When one drop collides with another, the protruding crystals can pierce the thin water film between the drops and be wetted by the oil in the other drop [99,100,101]. Walstra and co-workers [99,100] found that if there is sufficient liquid oil in the drops, the oil will flow around the crystal. The crystals within the drops form a solid network [102,103] that hinders the relaxation of the globules into a spherical shape. The partially coalesced droplets form an irregular shape (sometimes called a clump) [99,100]. Thivilliers-Arvis et al. [104] showed that the rate of partial coalescence depends on the size of the crystals and the extent to which they protrude from the drop surfaces.

Partial coalescence can also be observed in o/w emulsions containing solid particles that are completely wetted by the oil. Frostad et al. [105] measured the interaction forces between two solid-in-oil-droplets attached to two capillaries immersed in water. The magnitude of the forces measured between drops as they came into close contact were consistent with those predicted assuming that capillary bridges form between the drops [105]. Pawar et al. [106] observed coalescence between partially crystalline oil droplets, as the elastic modulus of the oil was varied by altering the concentration of fat crystals in the drops. At low particle concentrations, the drops behaved like weak gels with low elastic moduli. During coalescence the interfacial energy dominated the elastic energy and the merged drop relaxed into a spherical shape. The drop elastic modulus increased as the solids fraction increased. Pawar et al. [106] showed that at a critical particle concentration, the particle network elasticity balanced the Laplace driving force and prevented the merging drop from relaxing into a spherical shape.

Studart et al. [107] showed that when monodisperse drops partially coated with particles (formed using microchannel emulsification) are closely packed together they will coalesce into stable, non-spherical structures of two or more drops. They used electron microscopy imaging to show that there were dense layers of particles jammed together over the surface of the merged drops [107]. The attached particle layers on the merging drops make their surfaces sufficiently viscoelastic to increase the characteristic time for shape relaxation and (effectively) arrest coalescence. They argued that coalescence at one site on a drop surface does not lead to particle jamming across the whole oil-water interface. Providing patches of pristine drop surface remained exposed, a drop could undergo arrested coalescence with more than one adjacent droplet.

Pawar et al. [108] used a micromanipulation technique to make in situ observations of coalescence events between pairs of drops, of the same volume, each with a precisely known (fractional) surface coverage by particles (*C_1_* and *C_2_*). They observed that total coalescence dominates for initial surface coverage values of *C_1_* + *C_2_* < 1.43 [108]. Pairs of drops, each with a fractional surface coverage of 0.9, were stable to coalescence. Arrested coalescence was favoured at 1.43 < *C_1_* + *C_2_* < 1.8. Under these conditions, the combined surface area occupied by particles was higher than the interfacial area that would form by complete coalescence of the drops (Figure 5b) [108]. 

Morse et al. [109] visualised arrested coalescence between pairs of oil drops partially coated by polymer nanoparticles, providing cross-linker was present in one of the drops. They found that holding the drops in contact for about 60 s, and then decompressing them, led to arrested coalescence. Morse et al. [109] argued that the interfacial particles in the contact area become cross-linked and that moving the drops apart disrupts the cross-linked particles sufficiently to expose pristine drop surface and promote coalescence between the drops. Deformation of the drop surfaces caused by the separation of the drops may have also contributed [110].

**Figure 7:**
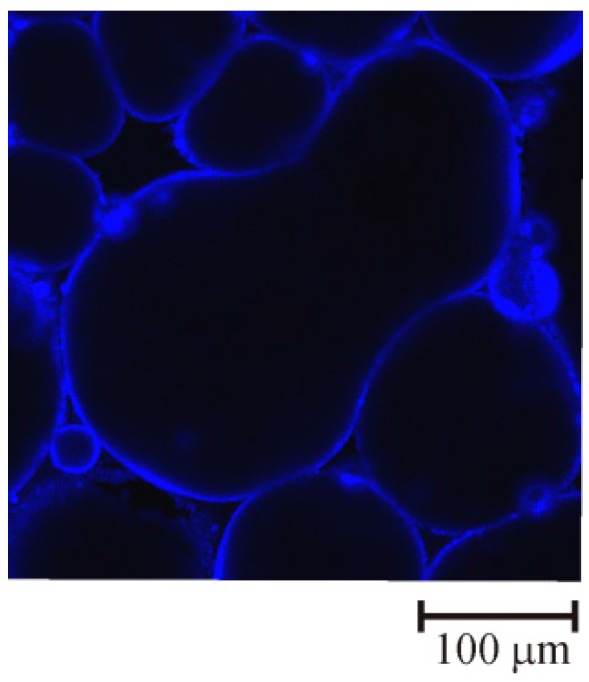
Confocal fluorescence image of the particles shells in an unstable, compressed Pickering emulsion showing a coalesced droplet fused into a doublet shape. Adapted from [111] with permission from the Royal Society of Chemistry.

Whitby et al. [111] found evidence of arrested coalescence in bulk Pickering emulsions. They used confocal fluorescence microscopy to visualise droplet packing in o/w emulsions stabilised by silanised silica particles as the emulsions were compressed. At the volume fraction where the emulsions started to break down, the drops increased in size, with some forming arrested shapes, as shown in Figure 7 [111]. Tan et al. [112] showed that arrested coalescence in emulsions can be triggered by Ostwald ripening of nanoparticles coating the drop surfaces. They studied paraffin-in-water emulsions coated with freshly precipitated Mg(OH)_2_ nanoparticles. Emulsions stored at temperatures higher than 80 °C destabilised [112]. A fraction of the drop population coalesced into non-spherical drop shapes. They argued that large precipitated nanoparticles grow in size at the expense of smaller nanoparticles at the elevated temperatures. This reduces the density of the particle coverage on the drop surfaces and triggers arrested coalescence of the drops. 

Whitby and Krebsz [64] investigated the rheology of concentrated oil-in-water emulsions stabilised by silanised fumed silica nanoparticles. These silica particles were shown to aggregate and form weak particle gels at high concentrations (0.5 M NaCl) of salt. Reducing the salt concentration in the emulsions increased the repulsive interactions between the interfacial particles. They found that this minimised the contribution of the particle layer tension to the interfacial energy of the drops [64]. Applying a stress on the order of the Laplace pressure destabilised the emulsions. Whitby and Krebsz [64] observed arrested coalescence for some drop pairs. They also observed that some coalesced drop surfaces were buckled and attributed this to the attached particles making the surfaces of merging drops solid-like in response to compression [64].

## 5. Harnessing Destabilisation to Fabricate Useful Materials

The developments in our understanding of how interfacial particles affect droplet (and bubble) destabilisation have renewed interest in emulsion phase inversion. This is the key process by which emulsions are transformed into new materials. Phase inversion of Pickering emulsions and foams has produced a wide range of new structures. For example, Binks and Murakami [113] formed powders of particle-stabilised water drops-in-air by inverting the curvature of the air-water surfaces in Pickering foams. Water drops coated with hydrophobic particles roll on solid surfaces like hard spheres [113]. They deform and break open under compressive stress [94]. The powders are used as delivery vehicles for aqueous ingredients in cosmetic products [114].

**Figure 8:**
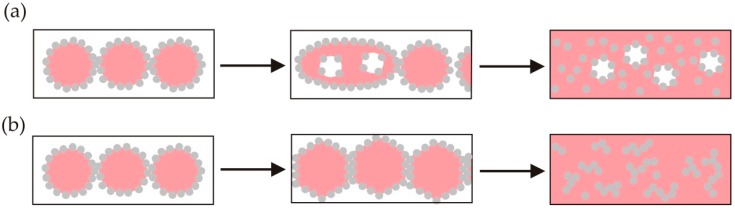
Pickering emulsions destabilise and catastrophically phase invert in response to an increase in their drop volume fraction. (**a**) Inversion may occur via the incorporation of drops of the continuous phase into drops of the dispersed phase as they coalesce in emulsions that are being sheared. Further shearing, or increases of the drop volume fraction, result in inversion of the dispersed and continuous phases in the emulsion; (**b**) Evaporation of the continuous phase from an emulsion can increase the volume fraction of the drops above the maximum fraction at which they can closely pack without distortion. Further evaporation causes the drops to coalesce together into a continuous liquid film and the particles to become completely immersed in the liquid.

Coalescence plays a central role in phase inversion. An emulsion of oil drops in water that is being agitated may suddenly transform into an emulsion of water drops in oil in response to a change in the particle wettability, or an increase in the drop volume fraction, as illustrated in Figure 8. The former process is called transitional phase inversion. The latter process is catastrophic. It is achieved by evaporation of the continuous phase, or by pumping the emulsion through narrow spaces. The properties of materials formed by phase inversion are determined by the structural changes that occur as the drops squeeze together and coalesce. 

New structures are made by manipulating coalescence events in Pickering emulsions at compositions close to phase inversion. For example, Binks and Whitby [115] observed that multiple emulsions form at conditions near catastrophic phase inversion. They proposed that coalescence dominates emulsion formation at compositions where the fraction of the dispersed phase is high [115]. Multiple drops form due to drops of the continuous phase being incorporated into drops of the dispersed phase as they coalesce together (Figure 8a) [115]. The main condition for stabilising multiple emulsions is to stabilise interfaces with both types curvature. Clegg et al. [116] recently reviewed the approaches to making multiple emulsions in the presence of a single type of particle. They argued that the wettability of a fraction of the particle surfaces must be modified by adsorption of air or components from the oil phase.

Monodisperse emulsions can be changed by using a step-wise process to make Pickering emulsions at compositions close to phase inversion. Binks and Rodrigues [117] found that increasing the oil volume fraction in an oil-in-water emulsion at a fixed particle concentration increases the average drop diameter, while reducing the polydispersity of the drop size distribution significantly. This approach forms monodisperse Pickering emulsions by exploiting the limited coalescence that occurs as a large excess of oil-water interface is formed in the presence of a small amount of particles. Direct and inverse emulsions have been fabricated in this way, with *r_d_* ranging from micrometres to millimetres [97,118].

Limited coalescence can also be manipulated to form emulsions at drop volume fractions above the fraction at which drops can be hexagonally close packed without distortion. The highly concentrated emulsions formed are known as high internal phase Pickering emulsions (HIPE). The drop volume fractions in HIPE are so high that they support their own weight and resist mechanical shear. Zang and Clegg [119] investigated the formation of water-in-oil HIPE from mixtures of toluene, water and silanised fumed silica nanoparticles by mechanical mixing. They proposed that HIPE form under conditions where the oil (dispersed) phase is sufficiently viscous to resist being fragmented by shearing and hence inversion [119]. The viscosity of the oil is affected by the particle concentration, shear rate and shearing time. HIPE have been used as templates for making macroporous solids when the liquid phases in the emulsion are removed by evaporation [20,120].

Adelmann et al. [121] reported making organogels by drying dilute oil-in-water Pickering emulsions. The oil phase in the emulsions transformed into a soft, plastic material (an organogel) as the water was removed. Whitby et al. [24] showed that catastrophic phase inversion of o/w Pickering emulsions during evaporation drives the assembly of nanoparticles into network structures within the non-volatile solvent (oil), as depicted in Fig 8b. The elasticity of the oil increases as the volume fraction of the particles in the oil increases. The scaling behavior of the elasticity is consistent with models of the particle networks in the oil as rigid chains connected by permanent, fixed cross-links [24]. 

Hijnen and Clegg [23] showed that organogels with cellular networks of particles are obtained by evaporating emulsions of partially miscible liquids. They found that closely-packed, polyhedral shapes formed by the drops as they squeezed together were retained in the particle network structure. Their approach utilised the buckling and cracking of particle-laden surfaces observed in emulsions as the two liquid phases mix [57]. Hijnen and Clegg [23] argued that this allowed defects to form in the interfacial layer of particles as the volatile liquid evaporated, but prevented complete rupture. This meant that network structure was not destroyed when the particles became fully wetted by the non-volatile solvent [23].

Oil-in-water Pickering emulsions can be dried to make powders containing encapsulated oil drops. This is achieved by spray drying the emulsions to rapidly remove the water and leave behind powdered solid containing oil. Simovic et al. [122] spray dried mixtures of oil drops (*r_d_* = 200 nm) and silica nanoparticles (*r_p_* = 7 nm) to form micrometre-sized particles of a porous silica matrix encapsulating the oil. Adelmann et al. [121] formed oil powders which did not leak oil for several months by spray drying emulsions with drops (*r_d_* = 10 μm) stabilised by fumed silica nanoparticles (*r_p_* = 30 nm). The powder granules were irregular, fractal-like aggregates, each consisting of several oil drops compressed together. They argued that the particles had sintered together, since the powders could not be re-dispersed in water [121]. 

The key to producing spray-dried powders that flow freely is controlling the radial distribution of components in the macroscopic droplets of emulsion that are injected into the drier. The structure of the granules formed depends on how the diffusion flux of each component compares to the radial velocity of the shrinking droplet surface [123]. Whitby et al. [124] made powders that could be re-dispersed into particle-coated drops by controlling the number of excess particles present in the emulsions and the extent of flocculation between the excess particles and the particle-stabilised drops. 

Bicontinuous materials can be derived from Pickering emulsions undergoing transitional phase inversion. For example, Binks [21] obtained porous solids with bicontinuous-like structures by drying emulsions with compositions close to that required for transitional inversion. Evaporating the oil and water from the emulsions produced solids consisting of interconnected pores that were worm-like in shape. Binks [21] speculated that bicontinuous domains of oil and water formed as the emulsions broke down, since interfaces with both types of curvature could be stabilised by the particles. Direct evidence for the formation of interfaces with zero net curvature in Pickering emulsions at conditions close to inversion was recently obtained. Destribats et al. [125] visualised the interfacial structure in emulsions as the particle wettability was altered systematically. They observed that particles with near neutral wettability show a bimodal distribution of contact angles [125]. The particles formed one of two different contact angles at the oil-water interface, with an average value close to 90°. 

Clegg et al. [57] took advantage of this behaviour to stabilise bicontinuous gels (bijels) made from mixtures of partially miscible liquids. The bicontinuous interface formed in a binary liquid as it demixes via spinodal decomposition can be arrested using particles that are neutrally wetted by both liquids. White et al. [126] recently demonstrated that bicontinuous domains form in mixtures of water, 2,6-lutidine and silanised silica nanoparticles (*r_p_* ~ 400 nm) at compositions near where the mixtures invert from lutidine drop-in-water to water drop-in-lutidine morphologies.

## 6. Conclusions

Pickering emulsions are metastable colloidal systems. Predicting the lifetime of a Pickering emulsion and how it will break down remains challenging. The stability of an emulsion depends on its composition and the external stresses applied to it during storage and handling. The findings we have reviewed indicate that irreversible changes to Pickering emulsions occur through three mechanisms; particle detachment, mass transfer between the liquid phases and drop coalescence.

Particle detachment from drop surfaces is caused by, for example, adsorbing surfactant on the particle surfaces to amplify their wettability by the external phase. Detachment also occurs under conditions where the particles are strongly flocculated. We suggest that this mechanism will be used as a tool for breaking Pickering emulsions under conditions where the application of physical stress (like heat, shear, magnetic or electrical fields) is undesirable. Akartuna et al. [127], for example, developed a strategy for merging surfactant-stabilised drops on microfluidic chips by chemically inducing pairs of drops to adhere and coalesce.

Mass transfer between the liquid phases in a Pickering emulsion can make the drops shrink and compress together the particles attached to the drop surfaces. The interfacial particles act collectively like a thin, solid shell and buckle under the applied stress. Manipulating the drops in Pickering emulsions into non-spherical shapes in this way is an avenue for fabricating large volumes of non-spherical colloids.

Mass transfer by Ostwald ripening causes larger drops to grow in size. Expansion of particle-laden interfaces may expose pristine drop surfaces and trigger coalescence events in Pickering emulsions. Coalescence is suppressed by adding excess particles to the emulsion. We propose that the ability to disrupt the particle layers coating drops in a transient fashion means it should be possible to fuse together drops of different liquids by destabilising mixtures of Pickering emulsions of different compositions. Fryd and Mason [128] exploited the analogous self-limiting fusion behavior in surfactant-stabilised emulsions to create large volumes of drops with multiple compartments that could encapsulate ingredients of different solubility.

Manipulating drop coalescence is critical to controlling the properties of materials formed by phase inversion. Coalescence between pairs of particle-coated drops due to changes in the surface coverage, the particle wettability or by physical stress has been studied. We think that the next step is to probe coalescence dynamics in bulk emulsions. Feng et al. [129] designed temperature-sensitive surfactants for controlling film formation by a surfactant-stabilised emulsion after visualising how the spatial arrangement of drops influenced phase inversion in the emulsion. Taking a similar approach to characterising how coalescence events propagate in Pickering emulsions will advance the design and performance of products based on the emulsions.
